# Pilot clinical trial and phenotypic analysis in chemotherapy-pretreated, metastatic triple-negative breast cancer patients treated with oral TAK-228 and TAK-117 (PIKTOR) to increase DNA damage repair deficiency followed by cisplatin and nab paclitaxel

**DOI:** 10.1186/s40364-023-00511-7

**Published:** 2023-07-25

**Authors:** Jessica D. Lang, Tuong Vi V. Nguyen, Maren K. Levin, Page E. Blas, Heather L. Williams, Esther San Roman Rodriguez, Natalia Briones, Claudius Mueller, William Selleck, Sarah Moore, Victoria L. Zismann, William P.D. Hendricks, Virginia Espina, Joyce O’Shaughnessy

**Affiliations:** 1 The Translational Genomics Research Institute (TGen), Integrated Cancer Genomics Division, Phoenix, AZ 85004 USA; 2 Department of Pathology and Laboratory Medicine, Center for Human Genomics and Precision Medicine, University of Wisconsin-Madison, Madison, WI 53705 USA; 3 Baylor Scott & White Research Institute, Dallas, TX 75246 USA; 4 Center for Applied Proteomics and Molecular Medicine, George Mason University, Manassas, VA 22030 USA; 5 Baylor University Medical Center, Texas Oncology, 3410 Worth Street, Suite 400, Dallas, TX 75246 USA

**Keywords:** Triple-negative breast cancer, Targeted therapy, Cell signaling, Genomics, Proteomics

## Abstract

**Background:**

A subset of triple-negative breast cancers (TNBCs) have homologous recombination deficiency with upregulation of compensatory DNA repair pathways. PIKTOR, a combination of TAK-228 (TORC1/2 inhibitor) and TAK-117 (PI3Kα inhibitor), is hypothesized to increase genomic instability and increase DNA damage repair (DDR) deficiency, leading to increased sensitivity to DNA-damaging chemotherapy and to immune checkpoint blockade inhibitors.

**Methods:**

10 metastatic TNBC patients received 4 mg TAK-228 and 200 mg TAK-117 (PIKTOR) orally each day for 3 days followed by 4 days off, weekly, until disease progression (PD), followed by intravenous cisplatin 75 mg/m^2^ plus nab paclitaxel 220 mg/m^2^ every 3 weeks for up to 6 cycles. Patients received subsequent treatment with pembrolizumab and/or chemotherapy. Primary endpoints were objective response rate with cisplatin/nab paclitaxel and safety. Biopsies of a metastatic lesion were collected prior to and at PD on PIKTOR. Whole exome and RNA-sequencing and reverse phase protein arrays (RPPA) were used to phenotype tumors pre- and post-PIKTOR for alterations in DDR, proliferation, and immune response.

**Results:**

With cisplatin/nab paclitaxel (cis/nab pac) therapy post PIKTOR, 3 patients had clinical benefit (1 partial response (PR) and 2 stable disease (SD) ≥ 6 months) and continued to have durable benefit in progression-free survival with pembrolizumab post-cis/nab pac for 1.2, 2, and 3.6 years. Their post-PIKTOR metastatic tissue displayed decreased mismatch repair (MMR), increased tumor mutation burden, and significantly lower levels of 53BP1, DAG Lipase β, GCN2, AKT Ser473, and PKCzeta Thr410/403 compared to pre-PIKTOR tumor tissue.

**Conclusions:**

Priming patients’ chemotherapy-pretreated metastatic TNBC with PIKTOR led to very prolonged response/disease control with subsequent cis/nab pac, followed by pembrolizumab, in 3 of 10 treated patients. Our multi-omics approach revealed a higher number of genomic alterations, reductions in MMR, and alterations in immune and stress response pathways post-PIKTOR in patients who had durable responses.

**Trial Registration:**

This clinical trial was registered on June 21, 2017, at ClinicalTrials.gov using identifier NCT03193853.

**Supplementary Information:**

The online version contains supplementary material available at 10.1186/s40364-023-00511-7.

## Statement of Translational Relevance

A subset of triple-negative breast cancers (TNBCs) have homologous recombination deficiency with upregulation of compensatory DNA repair pathways. We observed 3 patients with pretreated metastatic TNBC that had prolonged progression-free survival > 1 year with the immune checkpoint inhibitor, pembrolizumab, following sequential treatment with PIKTOR followed by cis/nab pac. These patients tended to have higher copy number alterations and/or tumor mutation burden in their metastatic disease after PIKTOR treatment, loss of DNA damage signatures, and increased expression of PD-1/PD-L1 pathway genes, with significant decreases in DNA damage response, stress and proliferation proteins. Their metastatic lymph node disease may have been primed or altered by the antecedent PIKTOR and/or cis/nab pac therapy. The results should be interpreted prudently due to the small sample size. However, this proof of concept study supports pursing larger, multi-site studies for PI3K and TORC1/2 and immune checkpoint blockade in metastatic TNBC.

## Background

Triple-negative breast cancer (TNBC) is an aggressive breast cancer subtype characterized by high mutational burden and high proliferation [1]. Up to 70% of TNBCs demonstrate high homologous recombination deficiency (HRD), as evidenced by HRD scores > 42 [2]. HRD impairs cancer cells’ ability to repair DNA damage inflicted by radiation or chemotherapy. HRD has been associated with improved outcomes in many cancers. HRD has been exploited as a therapeutic target because of demonstrated synthetic lethality in the context of agents such as PARP inhibitors that impair DNA damage repair (DDR) [3]. Inducing HRD with “priming agents” to render DNA damaging drugs and immune checkpoint inhibitors more effective has been a recent area of focus in the cancer field.

HRD can occur through BRCA1/2 mutation, methylation/silencing of *Fanconi Anemia* (*FA*) genes [4], loss of CDK1 activity [5], loss of Rad51-dependent foci formation [6], *TP53* mutation [7], and/or PI3K inhibition, among others [8]. TNBC is also reported to be associated with increased EGFR expression, a known effector of the PI3K/AKT/mTOR pathway, and upregulation of DNA repair via the non-homologous end joining (NHEJ) pathway [9, 10]. NHEJ is an error prone, template-independent pathway dominant throughout the cell cycle [11]. A key nuclear enzyme that orchestrates NHEJ is DNA-Dependent Protein Kinase catalytic subunit (DNA-PKcs), a member of the PI3K super-family [12]. Further, activating mutations in *PIK3CA, AKT1*, and *mTOR* are frequent in TNBC [13, 14], and PI3K-AKT-mTORC1 signaling can also be activated by DNA damage (reviewed in [15]). Loss of PTEN function in TNBC further leads to activation of AKT and the PI3K pathway [16].

Serine/threonine kinase mechanistic target of rapamycin (mTOR) is a downstream effector of the PI3K pathway and key component of two protein complexes, mTORC1 and mTORC2, which have distinct cellular functions. In preclinical studies, the dual inhibition of mTORC1 and mTORC2 decreased DDR and also decreased the activity of AKT [17]. This dual inhibition allowed for the partial re-sensitization of platinum-resistant ovarian cancer cells both in vitro and in vivo to platinum chemotherapy, as compared to single inhibition of mTORC1. They later found that this dual inhibition disrupts the protein translation of DNA damage repair genes including RAD51C, RAD17, POLQ, and POLB [18]. Blockade of the mTOR complexes may be a novel strategy to sensitize breast cancers to DNA damaging agents [17, 19].

TAK-228 (formerly INK128 and MLN0128) is an investigational, highly selective, orally bioavailable adenosine 5’ triphosphate (ATP)-competitive inhibitor of mTOR. TAK-228 targets both mTORC1 and mTORC2. TAK-117 (formerly MLN1117/INK1117) is an investigational, orally available, selective small molecule inhibitor of the Class I phosphoinositide 3-kinase (PI3K) alpha isoform (PI3Kα). This combination, referred to as “PIKTOR”, is hypothesized to achieve greater inhibition of the PI3K/AKT pathway than either agent alone [20]. Indeed, synergistic effects of TAK-228 and TAK-117 at their respective IC_50_s have been shown to reduce PI3K/AKT/mTOR (PAM) pathway activation, activate autophagy, and induce cell cycle arrest [21]. Inhibition of PI3K has been shown to induce an HRD-like state in BRCA-wildtype TNBC [8, 22]. mTOR inhibitors similarly induce HRD in TNBC [23]. Dual AKT/mTORC1 targeting in preclinical models impedes double-strand break repair and primes cancer cells for sensitivity to subsequent DNA damaging therapy [24]. Successful inhibition of the PAM pathway following selective inhibition of PI3Kα and mTORC1/mTORC2 has been shown to sensitize breast cancers to taxane or platinum-based therapies [25].

Several preclinical studies have shown that a variety of dual PI3K/mTOR inhibitors are synergistic with cisplatin in triple-negative breast cancer preclinical models [26–30]. One clinical case study of a patient with refractory metastatic triple negative breast cancer (mTNBC) demonstrated a switch from high EGFR expression and PI3K pathway activation to a loss of EGFR and PI3K activation and increased MAPK pathway activation after treatment with single agent BEZ-235, a PI3K/mTOR, ATM, ATR, and DNA-PKcs inhibitor. Upon disease progression, the patient was treated with 6 cycles of combined cisplatin and nab paclitaxel, developing a durable complete response [31].

PI3K inhibitors have also demonstrated reduced PD-L1 expression in tumors in triple-negative breast cancer, allowing enhanced detection by CD8 + T-cells [32]. A recent pre-clinical study shows promise of combination of PI3K inhibitors with immune checkpoint inhibitors [33]. Synergy with immune checkpoint inhibitors was not observed with a PI3K-alpha inhibitor but was observed with pan-PI3K and PI3K/mTOR inhibition. The same group previously observed that PI3K-gamma inhibition in CD8 + T-cells reduced triple-negative negative breast cancer growth in immune-intact xenograft models [34]. Therefore, we believe that a more complete inhibition of the PI3K and TORC1/2 pathway will increase HRD more than mTOR inhibition alone, sensitizing triple negative breast cancers to DNA damaging cisplatin and immune checkpoint inhibitors.

We investigated genomic and proteomic alterations in metastatic tissues for patients with mTNBC treated with the combination of TAK-228 and TAK-117 (PIKTOR) followed by cisplatin and nab paclitaxel (cis/nab pac) chemotherapy. This study evaluated whether PIKTOR increases genomic instability and increases DDR deficiency in patients’ metastatic tissue, leading to increased sensitivity to DNA damaging chemotherapy and immune checkpoint inhibitor therapy. We observed in our study that 3 patients with pretreated metastatic TNBC had prolonged progression-free survival > 1 year with the immune checkpoint inhibitor, pembrolizumab, following sequential treatment with PIKTOR followed by cis/nab pac. These patients tended to have higher copy number alterations and/or tumor mutation burden in their metastatic disease after PIKTOR treatment, loss of DNA damage repair deficiency signatures, and increased expression of PD-1/PD-L1 pathway genes. These 3 patients also had significantly lower levels of 5 proteins involved in DNA damage response, stress response, and proliferation.

## Patients and methods

### Study patients

Following Baylor University Medical Center IRB-approved informed consent, 10 female patients aged ≥ 18 years with metastatic TNBC were treated with PIKTOR, then with cis/nab pac at disease progression in this pilot, proof-of-concept trial. Key eligibility criteria included no more than three prior chemotherapy regimens for metastatic disease, ECOG PS 0–2, and metastatic disease amenable to core needle biopsy, and adequate hematologic, liver, and renal function.

### Study design

**Fig. 1:**
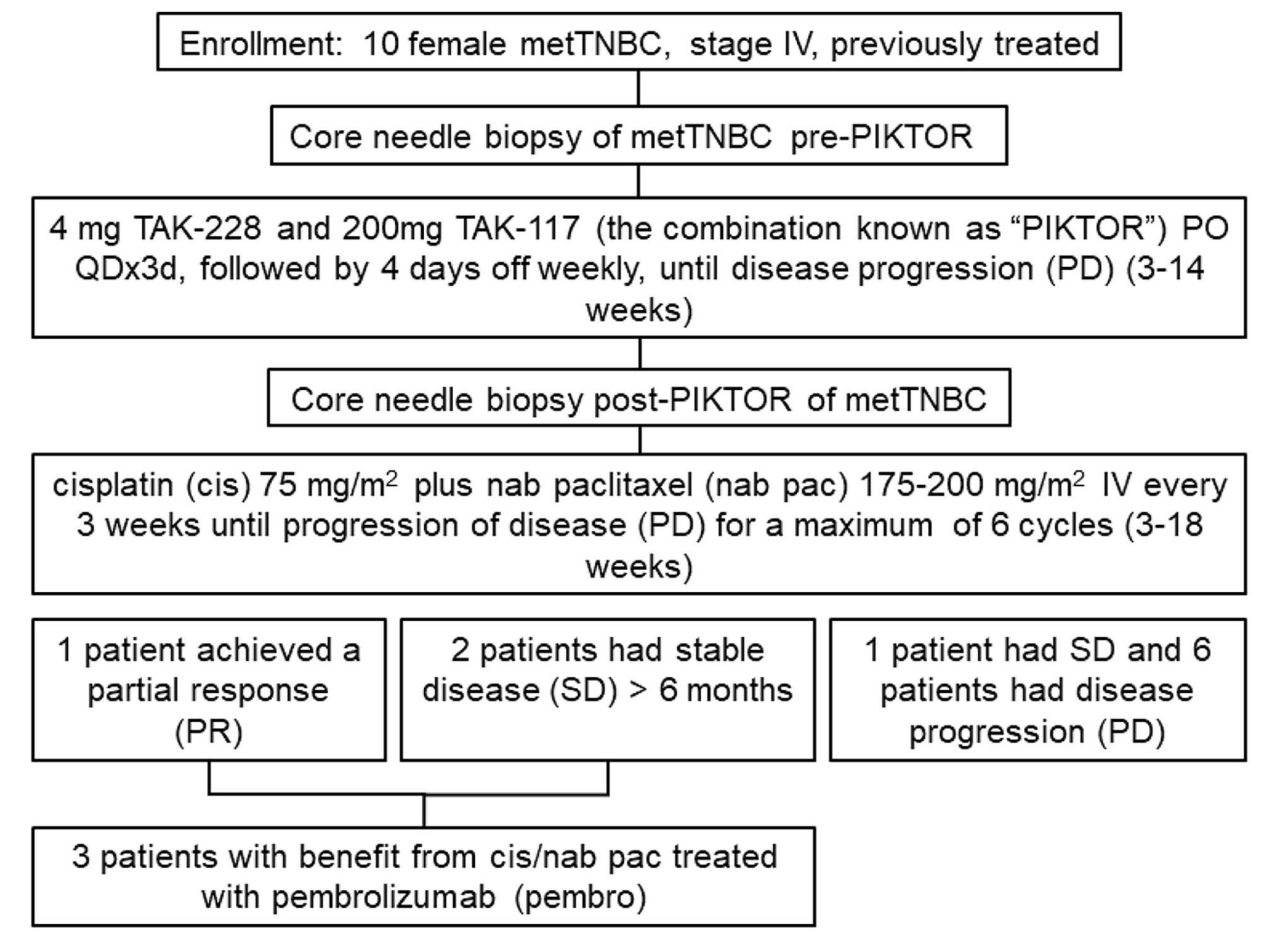
Trial consort diagram

This was a pilot, single center, open-label study (NCT03193853), and was carried out in accordance with the principles of Good Clinical Practice and the Declaration of Helsinki. Patients received 4 mg TAK-228 PO and 200 mg TAK-117 PO (PIKTOR) both QD for 3 days on, followed by 4 days off, weekly until disease progression (PD), followed by 75 mg/m^2^ cisplatin (cis) plus 175–200 mg/m^2^ nab paclitaxel (nab pac) IV every 3 weeks until progression of disease (PD) or for a maximum of 6 cycles. Patients were then treated with pembrolizumab, alone or in combination with chemotherapy, regardless of PD-L1 status, or with chemotherapy or targeted therapy if not a candidate for immunotherapy based on disease course (Fig. 1).

Blood samples and a research biopsy of a metastatic lesion were collected prior to initiation of PIKTOR and again upon disease progression on PIKTOR. Blood samples were collected in 10mL purple-top EDTA hematology tubes, processed within 1 h into plasma and buffy coat, and stored at -80 °C. Buffy coat was used for germline whole exome sequencing as described below. Biopsies were snap frozen in a cryovial by submerging into liquid nitrogen and stored at -80 °C prior to analysis with whole exome sequencing, RNA sequencing, and proteomics as described below. Patient demographics and tumor characteristics are summarized in Table 1.

**Table 1:** Patient demographics and tumor characteristics

Patients								Biomarkers	Treatment on Study			Post-Study Treatment	
Patient ID	Age at Study Entry	Ethnicity	Sites of Metastatic Disease	Prior Therapies (neo/adjuvant)	Prior Therapies (metastatic)	Pre-PIKTOR Biopsy Site	Post-PIKTOR Biopsy Site	Standard of Care Germline Tumor/Blood NGS and IHC Results	Total weeks on PIKTOR	Total Weeks on Cis/Nab Pac	Response to Cis/Nab Pac	Treatment	Duration (weeks)
001	40	White	Internal mammary LN, abdominal LN, bone, brain	Cisplatin Docetaxel + Cyclophosphamide	Gemcitabine + Carploplatin Xeloda	Inguinal LN	Same as pre-PIKTOR site	G: BRCA1 +; BRCA2 -	7	16	SD	Pembrolizumab	106
								S: NOTCH1; TP53; MYC; FGFR2; CIC Amplification of PDGFRA					
								IHC: PD-L1 Negative; 22C3: PD-L1 Negative					
002	57	White	Chest wall, mediastinal LN, lung	Doxorubicin + Cyclophosphamide + Paclitaxel Carboplatin + Capecitabine	N/A	Lung	Axillary LN	G: BRCA1/2 -	9	3	PD	Entrectinib	5
								S: TP53; MYO5A NTRK3; PAK1; CDKN2A; CDKN2B Amplification of C11ORF30 (EMSY); PIK3CA					
004	38	White	Mediastinum LN, lung	Doxorubicin + Cyclophosphamide + Paclitaxel Carboplatin Capecitabine	N/A	Cervical LN	Supraclavicular LN	G: Panel Germline Testing Negative	10	17	PR	Paclitaxel + Bevacizumab + Pembrolizumab	18
								S: PTEN; LZTR1; NOTCH2; TP53; AR; FGFR1; PIK3CA; MET MTAP; ARID2 Amplification of ZNF217; PIK3CA; RAF1; KRAS; CCND2; CCND1; MET; BRAF; FGFR2; PDGFRA; KIT; CDK6; CCNE1					
								IHC: PD-L1 Negative					
005	58	White	Axilla LN, lung	Doxorubicin + Cyclophosphamide + Paclitaxel	N/A	Lung	Same as pre-PIKTOR site	G: BRCA1 +; other Panel Germline Testing Negative	5	9	PD	Eribulin + Pembrolizumab	11
								S: TP53					
								PD-L1 Negative					
006	68	White	Internal mammary LN, supraclavicular LN	Doxorubicin + Cyclophosphamide + Paclitaxel Carboplatin + Capecitabine	Letrozole + Xeloda Faslodex + Palbociclib	Supraclavicular LN	Same as pre-PIKTOR site	G: BRCA1/2 -; MUTYH	11	6	PD	Pembrolizumab	190
								S: TP53; INPP4B; MLL3; MEF2B; Amplification of CDK4					
								IHC: PD-L1 Positive					
008	39	African American	Axillary LN, supraclavicular LN, thoracic LN, internal mammary LN	Doxorubicin + Cyclophosphamide Carboplatin + Paclitaxel	Gemcitabine + Carboplatin	Axillary LN	Same as pre-PIKTOR site	G: Panel Germline Testing Negative	14	18	SD	Pembrolizumab	65
								S: PIK3CA; TP53; RB1;					
								IHC: P-L1 Negative, CPS 10, 22C3; PD-L1 Positive, CPS 11, 22C3					
009	48	White, Hispanic/ Latino	Lung, mediastinal LN	Docetaxel + Cyclophosphamide	N/A	Lung	Same as pre-PIKTOR site	G: Panel Germline Testing Negative	4	7	PD	Pembrolizumab	12
								S: PTEN; TP53; RB1; MLL2; CREBBP; MLL3 Amplification of FGFR1; PIK3CA; CCNE1; MYC					
								IHC: PD-L1 Negative					
010	63	White,	Supraclavicular LN, mediastinal LN, hilar LN	Doxorubicin + Cyclophosphamide + Paclitaxel	Taxol	Supraclavicular LN	Same as pre-PIKTOR site	G: Unknown	14	17	SD	N/A	N/A
								S: TP53; BAP1; PTEN; ETV6;					
								IHC: PD-L1 Negative					
011	42	Asian	Supraclavicular LN	Epirubicin + Docetaxel	Niraparib PARP inhibitor clinical trial Carboplatin + Gemzar Eribulin	Supraclavicular LN	Same as pre-PIKTOR site	G: BRCA1 +; other Panel Germline Testing Negative	7	3	PD	Paclitaxel + Bevacizumab + Capecitabine	5
								S: TP53; TCF7L2; INPP4B; BAP1 Amplification of FGFR2; MYC					
								IHC: PD-L1 Negative					
012	51	White	Paratracheal LN, lung	Doxorubicin + Cyclophosphamide + Paclitaxel	Carboplatin, Gemcitabine Abraxane + Tecentriq Pembrolizumab	Lung	Same as pre-PIKTOR site	G: Panel Germline Testing Negative	3	6	PD	Erubilin	7
								S: TP53					

Abbreviations: LN = lymph node; N/A = not applicable; PD = progression of disease; PR = partial response; SD = stable disease; Cis = Cisplatin; Nab Pac = Nab-Paclitaxel; G = Germline Mutations; S: Somatic Mutations; VUS: Variance of Unknown Significance; IHC = Immunohistochemistry; NGS = Next-generation sequencing;

Footnote: Patients 003 and 007 were screen fails and did not receive treatment; Patients 001 and 006 are still alive, Patient 001 is on currently on bevacizumab and Patient 006 is currently on pembrolizumab

### Study assessments

The primary objectives of this pilot clinical trial were to determine the objective response rate and safety associated with cis/nab pac following PIKTOR therapy. Secondary objectives were to determine duration of response and to assess mTNBC tissues obtained pre- and post-PIKTOR for homologous recombination proficiency and deficiency using next-generation sequencing and reverse phase protein array.

### Nucleic acid extraction and NGS library preparation and sequencing

Fresh frozen tumor biopsies were disrupted and homogenized with BulletBlender Gold tissue homogenizer (Next Advance) and divided for DNA and RNA extraction using DNeasy Blood & Tissue kit (Qiagen) and RNeasy kit (Qiagen), respectively, per manufacturer’s protocols. DNA was eluted into Qiagen’s AE buffer, and RNA was eluted into water.

Isolated DNA (200ng) was used to generate libraries for whole exome sequencing. DNA was fragmented to target peaks of 200 bp by sonication on a Covaris E220. DNA samples were prepared for next generation exome sequencing with SureSelect XT (Agilent), according to manufacturer’s protocol with the following modifications: Library amplification conditions: initial denaturation at 98 °C for 2 min, 8 cycles of 98 °C for 30 s, 60 °C for 30 s, and 72 °C for 60 s, then final extension at 72 °C for 5 min. Whole exome libraries were captured using Agilent SureSelect XT v7. The samples were sequenced on a NovaSeq6000 (paired end x 100 bp; Illumina) to an average depth of 352x ± 67x coverage.

Isolated RNA (750ng or total amount) was used to generate libraries for RNA-seq. RNA samples were prepared for next generation sequencing with KAPA stranded RNA HyperPrep Kit with RiboErase (Roche) with KAPA Unique Dual Index adapters (Roche), as per manufacturer’s protocol with the following modifications: fragmentation at 94 °C for 6 min; 10 cycles of library amplification; purifications with AMPure XP beads (Beckman Coulter Genomics). The libraries were sequenced on a single lane on a NovaSeq6000 (paired end x 100 bp; Illumina) to an average total reads of 1.94E + 08 ± 0.84E + 08 reads.

### Whole exome sequencing data analysis

Primary analysis was performed using the Translational Genomics Research Institute (TGen) Jetstream pipeline Phoenix (https://github.com/tgen/phoenix). BCL files were converted to FASTQs with bcl2fastq2 Conversion Software (Illumina). DNA FASTQs were aligned to the human reference genome (GRCh38) with BWA v0.7.17. Duplicates were marked using Samtools v1.10 markdup. BAMs were used for the identification of somatic variants (point mutations, insertions, deletions; SNVs), structural variants (SVs), and copy number variants (CNVs). Variant calling was performed using Strelka2 v2.9.10, Lancet v1.1.0, GATK v4.1.8.0 Mutect2, VarDictJava 1.7.0, and Octopus v0.603-beta. Final somatic SNVs were called by at least 2 out of 5 callers. CNVs were predicted with GATK v4.1.8.0 CNV. SVs were predicted using Manta v1.6.0 and Pairoscope 0.4.2. Somatic variants, SVs, and CNVs were annotated using Ensemble v98, then filtered by removing variants predicted to have no protein coding sequence consequence, as well as removal of genes flagged as LOWQC (https://github.com/tgen/GemDb/wiki/Filtering). Where known oncogenes and tumor suppressor genes are reported, variants were annotated against Catalog of Somatic Mutations in Cancer (COSMIC) gene list (v82) and filtered if gene does not occur in COSMIC database. cBioPortal’s Oncoprinter was used to generate oncoprint plots. Variant calls were confirmed by visual inspection of BAM and SEG files in Integrative Genomics Viewer v2.8.9. DNA damage repair gene list was generated using gene ontology terms for “DNA repair”, “chromatin remodeling”, and “methyltransferase”. Full list of genes is in Supplemental Table 1.

To extract DDR signatures from whole exome sequencing data, deconstructSigs v1.9 was run using output VCFs from the whole exome sequencing pipeline as described above. We first filtered variants to include only those identified by 2 out of 3 callers. The nature2019 signature was run, and presence or absence DDR-related signatures in resulting output was reported.

### RNA-Sequencing data analysis

Primary analysis was performed using the Translational Genomics Research Institute (TGen) Jetstream pipeline Phoenix (https://github.com/tgen/phoenix). BCL files were converted to FASTQs with bcl2fastq2 Conversion Software (Illumina). RNA FASTQs were aligned using STAR v2.7.5a. Gene expression estimates were performed using Salmon v1.2.1 (for TPM calculation) and STAR v2.7.5a GeneCounts quantMode / HTSeq v0.12.3 (for counts). DESeq2 v1.34.0 was used to determine differential gene expression changes between the specified groups using sequencing batch and patient ID as covariates. Results from all protein coding genes from DESeq2 results were analyzed with the use of QIAGEN IPA (QIAGEN Inc., https://digitalinsights.qiagen.com/IPA; run date: 02/26/2021), which was run with the following settings: log_2_FoldChange cutoff of +/- 1.2, p-value cutoff of 0.05, Ingenuity Knowledge Base (Genes Only) reference set. Canonical Pathway results from IPA were generated using default settings, and pathways where a Z-score could not be calculated were filtered out of analysis. Common pathway grouping (i.e., “immune pathways” and “PI3K related”) were manually curated.

### Proteomic analysis: laser capture microdissection (LCM)

Twenty core metastatic disease biopsies representing pre- and post-PIKTOR treatment specimens were embedded in Optimal Cutting Temperature (OCT) compound and sectioned (8 μm) onto plain glass microscope slides. Histomorphology was assessed with hematoxylin-eosin (H&E) staining following standard protocols [35].

Tumor and stromal cells were isolated as separate enriched cell populations using LCM (ArcturusXT or PixCell IIe instruments) in infrared capture mode as previously described [35]. Frozen tissue sections were fixed in 70% ethanol, stained with hematoxylin, dehydrated in graded ethanol and rinsed in xylene. The staining reagents contained protease inhibitors (CompleteMini tablets, Roche#11836153001). Microdissected cells captured on LCM caps (CapSure Macro cap, ThermoFisher #LCM0211) were lysed with protein extraction buffer (10% (v/v) Tris(2-carboxyethyl)phosphine (TCEP; Pierce #77720, Rockford, IL) in equal volumes of Tissue Protein Extraction Reagent (T-PER™, Pierce #78510) and Novex Tris-glycine 2X SDS buffer (Invitrogen #LC2676). Lysates were denatured at 95 °C for 5 min and stored at -80 °C prior to reverse phase protein array construction.

### Reverse phase protein arrays (RPPA) construction

RPPA were constructed with microdissected breast tissue lysates diluted to 0.25 µg/mL, commercial cell line lysates, and bovine serum albumin (BSA, Pierce #23209) [36–38]. Lysates were printed in technical replicates onto nitrocellulose coated glass slides (Oncyte Avid, Grace Bio-Labs), using an Aushon 2470 arrayer (Quanterix) equipped with 350 μm solid pins. SK-BR-3 nuclear extract (Santa Cruz Biotechnology sc-2134, breast adenocarcinoma), MCF7 + EGF + β-estradiol (Santa Cruz Biotechnology sc-24730, breast adenocarinoma), and BT-474 (ATCC HTB-20) cell lysates were printed on each array as quality control samples. BSA was printed in a calibration curve to quantify total protein per spot [37, 39] using Sypro Ruby protein blot stain (Invitrogen/Molecular Probes) per manufacturer’s directions and scanned using a Cy3 laser (Tecan Power Scanner).

### Immunostaining

Immunostaining was performed on a Dako Autostainer per manufacturer’s instructions (CSA kit and Genpoint kit, Agilent) [38, 40]. Each slide was incubated with a single primary antibody at room temperature for 30 min (Supplemental Table 2), and antibody specificity was confirmed by Western blotting as previously described [41]. The negative control slide was incubated with antibody diluent. Secondary antibody was goat anti-rabbit IgG H + L (1:10,000) (Vector Labs) or anti-mouse IgG (1:10, Agilent Dako CSA kit).

Spot (pixel) intensity with adjusted background correction was analyzed using ImageQuant v5.2 (Molecular Dynamic) [36, 39]. Data reduction algorithm (RASv16, http://capmm.gmu.edu/rpma-analysis-suite) was used to quantify and normalize the relative intensity value to the total protein/spot [38].

### Proteomic statistical analysis

Statistical significance was calculated using the Mann-Whitney test with the Holm-Bonferroni method to correct for multiple comparisons. Mean comparisons were performed using Wilcoxon Rank Sum due to small sample size. Potentially significant variables were selected based on non-corrected p-values. A p-value < 0.05 was used to indicate statistical significance. Heatmaps were created using unsupervised two-way hierarchical clustering (RStudio). Correlograms were prepared by calculating Spearman correlations between all protein endpoints for the indicated group of samples. All statistical calculations were performed using R 4.1.0 [42]. Violin plots and bar graphs were created with GraphPad Prism (v9.5.1).

## Results

### Clinical trial safety and efficacy of PIKTOR followed by cisplatin/nab paclitaxel

**Fig. 2:**
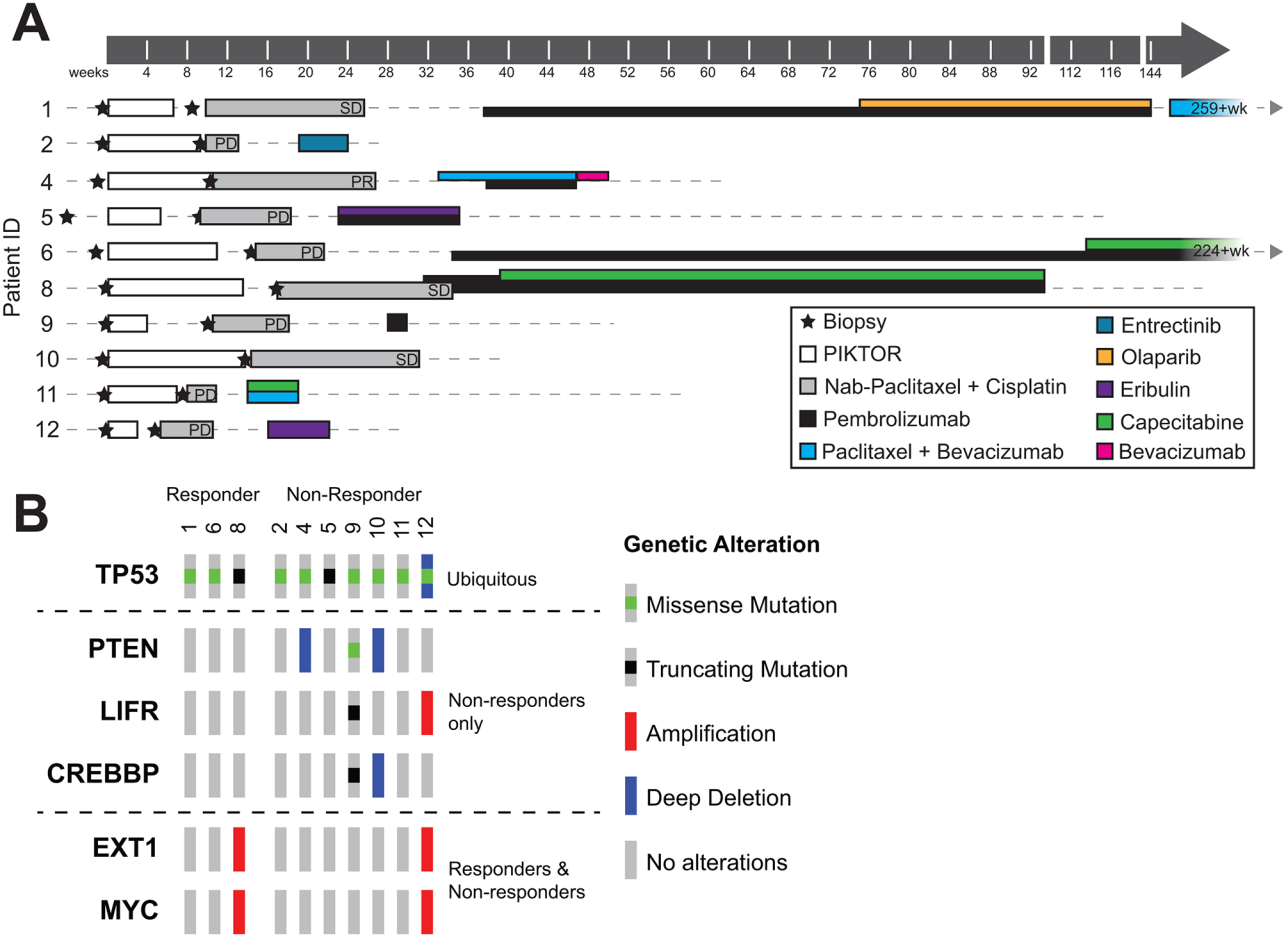
Clinical biopsy and treatment timeline and genetic characterization. **(A)** Clinical timeline of pre- and post-PIKTOR biopsies, time on PIKTOR treatment and follow-on therapies. Time 0 set at Day 1 of PIKTOR regimen. Arrows indicate survival at last follow up as indicated. **(B)** Oncoprint summarizing somatic mutations and copy number alterations in known oncogenic driver genes from COSMIC cancer gene consensus in pre-PIKTOR biopsies. Only recurrently altered genes are shown (see Supplemental Table 3 for full variant list). Patients are separated by long-term response, and genes are grouped by patterns of alteration in responders and non-responders

Between July 2017 and December 2018, a planned 10 patients with metastatic TNBC were enrolled from a single site to receive PIKTOR with subsequent cis/nab pac chemotherapy at disease progression (Figs. 1 and 2 A and Table 1). The median patient age was 49.5 years (range: 38–68). The median number of prior chemotherapy regimens was 3 (range: 1–5); 7 patients had prior carboplatin. Sites of pre-PIKTOR metastatic disease included lymph node (LN; n = 9), lung (n = 5), chest wall (n = 1), bone (n = 1), and brain (n = 1). All patients had measurable disease per RECIST 1.1 criteria. Tissue biopsies were obtained at baseline (pre-PIKTOR) and at the time of disease progression on trial (post-PIKTOR; Table 1).

The median time on PIKTOR for all patients prior to developing PD was 8 weeks (range: 3–14 weeks); the 3 patients considered durable “responders” had a median time on PIKTOR of 11 weeks (range: 7–14 weeks) and the 7 patients who did not show clinical benefit had a median time on PIKTOR of 7 weeks (range: 3–14 weeks). The median time between previous platinum/taxane exposure and study cis/nab pac for all patients was 58.5 weeks (range: 19–163 weeks) with a median time for the 3 durable “responders” of 101 weeks (range: 19–134 weeks) and a median time for the 7 “non-responders” of 48 weeks (range: 19–163 weeks). The median time for all patients on cis/nab pac was 8 weeks (range: 3–18 weeks). PIKTOR administration was generally well tolerated. PIKTOR-related adverse events (AEs) experienced in ≥ 30% of patients included: fatigue (90%); nausea (80%), diarrhea (60%), vomiting (40%), stomatitis (40%), hyperglycemia (30%), rash (30%), cough (30%), and chest pain (30%). Incidence and grade of cis/nab pac-related AEs were as expected and were not obviously increased or decreased post-PIKTOR therapy (Supplemental Table 6).

After progression on PIKTOR, and with subsequent cis/nab pac treatment, 1 patient had a partial response (PR), 2 patients had stable disease (SD) ≥ 6 months, 1 patient had SD for less than 6 months, and 6 patients had disease progression as best response (PD). The objective response rate was 10% and the clinical benefit rate was 30%. Three of 10 patients (patients 1, 6, and 8), each of whom had carboplatin-pretreated disease metastatic to lymph nodes, as well as in bone (n = 1), and whose pre-PIKTOR tumor biopsies were PD-L1-negative by IHC (n = 2) or PD-L1-positive (n = 1), had durable SD on single agent pembrolizumab following PIKTOR and cis/nab pac therapy for 106, 190 and 65 weeks, respectively. These patients were considered durable “responders” for the purpose of biomarker exploration in this study (Fig. 2A). Three additional patients received pembrolizumab alone or with chemotherapy following progression on cis/nab pac, but had less than 6 months of progression-free survival. Three other patients received chemotherapy or the NTRK inhibitor, entrectinib, because they were not deemed to be candidates for immunotherapy, but had rapid disease progression. The 10th patient did not receive subsequent therapy due to rapid disease progression and death.

### Somatic variant analysis of TNBC metastatic biopsies pre-treatment

The 3 patients (1,6,8) who had prolonged response to single agent pembrolizumab following PIKTOR and cis/nab pac therapy were considered responders. Their tumor and pre-treatment characteristics are summarized in Table 1. We hypothesized that these patients’ metastatic lymph node disease may have been primed or altered by the antecedent PIKTOR and/or cis/nab pac therapy to respond to pembrolizumab as it was unlikely their platinum-pretreated, PD-L1-negative (known to be the case in 2 of the 3 responders) disease would have benefited durably from cis/nab pab followed by pembrolizumab. The remaining 7 patients were considered non-responders as they did not have a durable response with cis/nab pac nor with their subsequent therapy. To determine whether these 3 durable responders had somatic variants in individual genes in common that might explain their benefit from pembrolizumab, copy number alterations and SNVs were compared between responders and non-responders. Recurrently altered COSMIC oncogenes and tumor suppressor genes are shown in Fig. 2B. *TP53* mutations were found in all 10 patients. No COSMIC oncogenes or tumor suppressors were unique to or were consistently mutated in the responders’ tumor tissues. *PTEN, LIFR*, and *CREBBP* alterations were found only in non-responders.

### PIKTOR treatment associated with decreased gene expression

To determine the effect of PIKTOR treatment on transcription, we performed differential expression analysis on pre- and post-PIKTOR biopsies (Supplemental Table 4). When the entire cohort of 10 patients was analyzed together, only 3 genes were significantly differentially expressed between pre- and post-PIKTOR biopsies: PKHD1L1, COL4A3, and DERL3 and each was significantly decreased. We further assessed cellular pathway alterations using Ingenuity Pathway Analysis (IPA), and similarly found that one pathway, GP6 signaling, had a p-value < 0.05, attributable to changes in expression of collagen genes after PIKTOR treatment (data not shown).

**Fig. 3:**
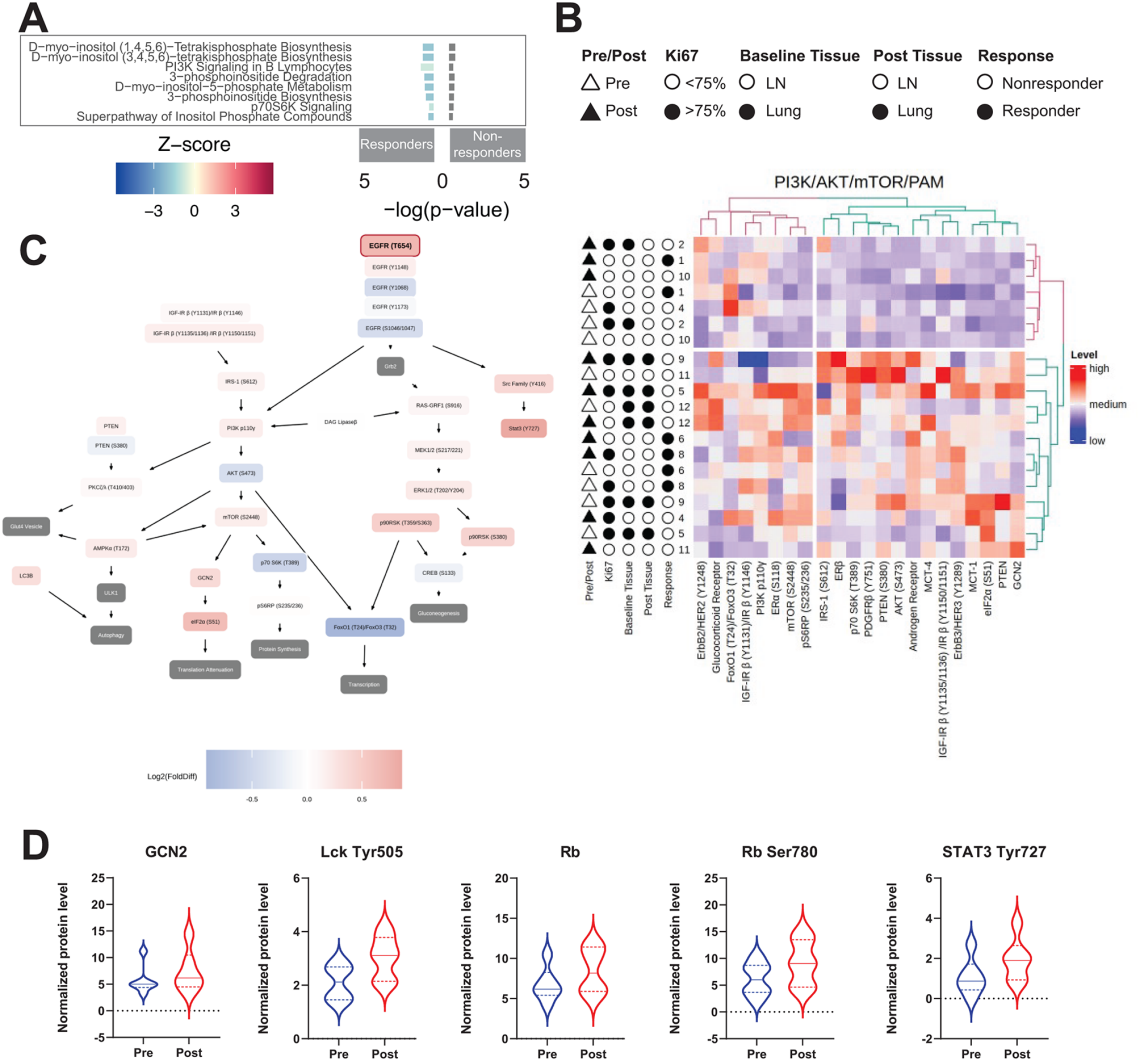
PI3K-AKT-mTOR pathway changes following PIKTOR treatment. **(A)** Pathway changes following PIKTOR treatment. Canonical pathway enrichment from differential gene expression results between pre- and post-PIKTOR biopsies. DESeq2 results from independent Responder and Non-responder analyses were input into Ingenuity Pathway Analysis, and results from the canonical pathway output is grouped by broader common pathways. Z-score is represented by color, and –log(p-value) is displayed by bar chart height, with values for responders extending to the left of the origin, and values for non-responders to the right. **(B)** Unsupervised hierarchical clustering levels of PI3K/AKT/mTOR (PAM) pathway proteins in microdissected tumors that metastasized to lymph node reveals heterogeneous signaling profiles. Pre and post samples are represented by white and black triangles, respectively. **(C)** Biological signaling pathway superimposed with log_2_ fold changes in average protein levels post-PIKTOR compared to pre-PIKTOR. Lymph node metastasis specimens only (n = 6). **(D)** Statistically significant proteins in cell stress, protein translation, T cell receptor activation, and cell cycle pathways were elevated in the post-PIKTOR treated patient specimens compared to pre-PIKTOR. Violin plots: solid line is group median, with quartiles (dashed lines)

To determine if specific pathways correlated with potential immune response priming with PIKTOR treatment, we separately analyzed responders and non-responders. With this we were able to detect many more statistically significant differential expression changes (Supplemental Table 4). For pathway analysis, we focused on patients whose pre- and post-PIKTOR biopsies were obtained from lymph nodes (N = 6), as this was the most common biopsy site, which avoided pathway signature expression differences due to different metastatic organ sites. Pathways related to the biosynthesis of PI3K pathway substrates or signaling of PI3K/AKT/mTOR did not pass our p-value filter but were detected in the IPA analysis (Fig. 3A, Supplemental Fig. 1). Responders had negative Z-scores indicating a reduced activation of these pathways with PIKTOR treatment. Non-responders had fewer differentially expressed genes in these pathways, and thus did not generate a Z-score. Pathways that showed decreased activation in responders post-PIKTOR included: D-myo-inositol (1,4,5,6)-Tetrakisphosphate Biosynthesis, D-myo-inositol (3,4,5,6)-Tetrakisphosphate Biosynthesis, PI3K Signaling in B Lymphocytes, 3-phosphoinositide Degradation, D-myo-inositol-5-phosphate Metabolism, 3-phosphoinositide Biosynthesis, p70S6K Signaling (downstream of AKT and mTOR), and Super pathway of Inositol Phosphate Compounds. While not statistically significant, these findings are consistent with more robust PI3K pathway inhibition by PIKTOR in the patients who responded durably to subsequent cis/nab pac followed by pembrolizumab.

### Proteomic cell signaling reveals modulation of stress response proteins in microdissected tumor

The genomic immune signatures we identified reflected the metastatic lymph node-tumor microenvironment within the lymph nodes that were biopsied. To assess functional cell signaling modulation in the metastatic tumor cells, we quantified cell signaling protein levels in microdissected tumor cells by reverse phase protein array (RPPA). Considering the small patient sample size (n = 10), we evaluated statistical significance without multiple comparisons correction to determine possible trends within the data that may highlight protein endpoints for further analysis. Protein levels pre- and post-PIKTOR were compared for all metastatic samples for both lung and lymph node metastatic sties. The post-PIKTOR samples revealed overall increased protein levels for Androgen Receptor (AR) (p < 0.05), AMPKα Thr172 (p < 0.037), EGFR Thr654 (p = 0.004), Estrogen Receptor alpha (ERα) Ser118 (p = 0.014), Retinoblastoma (Rb) (p = 0.009), and General Control Non-depressible 2 (GCN2) (p = 0.002) (Supplemental Fig. 2). Unsupervised hierarchical clustering of PI3K/AKT/mTOR signaling nodes (Fig. 3B) revealed heterogeneous protein expression within the entire cohort based on biopsy collection time (pre- vs. post-PIKTOR), metastatic site, Ki-67 positivity (above vs. below the 75% threshold), and treatment response. Two of the three patients in the responder group (6 and 8) were in the same cluster with higher levels of Androgen Receptor and phosphorylated IGF-1R β Y1150/1151, and lower levels of phosphorylated HER2 Y1248. To visualize PI3K-AKT-mTOR protein signaling pathway changes pre and post-PIKTOR treatment in the lymph node metastatic samples, we constructed a pathway map of the log_2_ fold changes in protein post-PIKTOR compared to pre-PIKTOR. Key signaling nodes in the PI3K pathway were lower post treatment (AKT Ser473, p70S6 Thr389, FOXO1/O3 T24/32) post-PIKTOR indicating functional suppression of signaling by PIKTOR (Fig. 3C).

To determine if the metastatic tissue site had an effect on cell signaling protein levels, we excluded sample 2 from the statistical analysis because the pre-PIKTOR metastatic tissue was lung and the post-PIKTOR tissue was lymph node. As in the entire cohort, AR, EGFR Thr654, ERα Ser118, GCN2, and Rb protein levels were higher in post-PIKTOR samples (Supplemental Fig. 3). Further analysis of only the microdissected tumor cells metastatic to lymph nodes (n = 6) showed higher levels of stress and immune signaling proteins (GCN2, Lck Tyr505, Rb, Rb Ser780, and Stat3 Tyr727) (Fig. 3D). These findings demonstrate pharmacodynamic effects expected with PI3K/mTOR pathway inhibition in TNBC and suggest that the dose and schedule of PIKTOR were adequate to induce functional stress responses.

### PIKTOR treatment resulted in increased copy number alterations and tumor mutation burden, particularly in responders

**Fig. 4:**
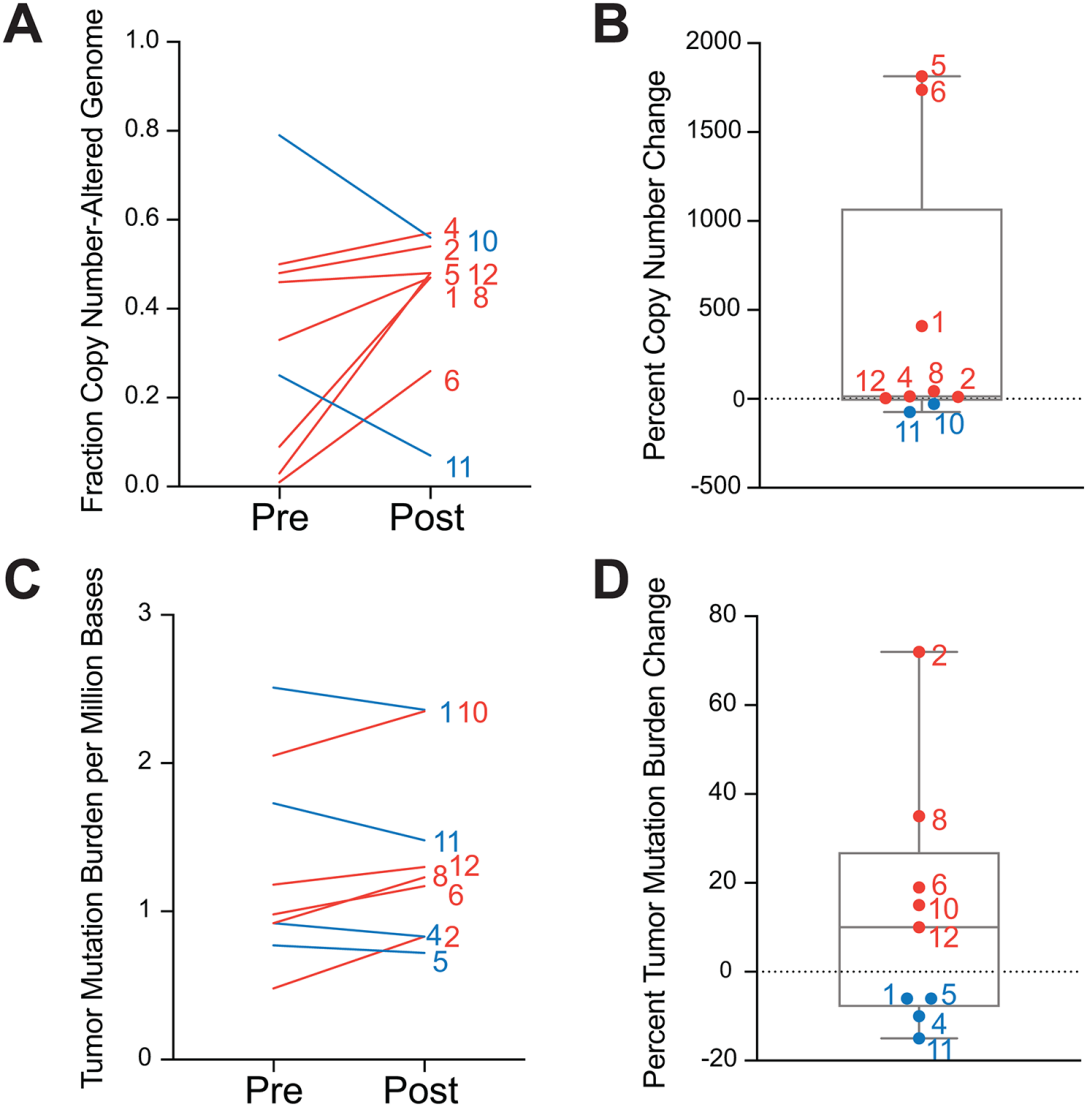
Changes in tumor mutation burden and copy number following PIKTOR treatment. **(A)** Individual fraction copy number-altered genome values for pre- and post- PIKTOR biopsies. Patient ID is labeled on right. **(B)** Percent change in fraction copy number-altered genome from **(A)**. Boxplot shows median and upper and lower quartiles, as well as the highest and lowest values represented by error bars. Each patient is labeled with their identifier. **(C)** Individual tumor mutation burden per million bases for pre- and post- PIKTOR biopsies. Patient ID is labeled on right. **(D)** Percent change tumor mutation burden from **(C)**. Boxplot shows median and upper and lower quartiles, as well as the highest and lowest values represented by error bars. Each patient is labeled with their identifier

The premise that PIKTOR would decrease DNA damage repair and make patients’ TNBC more sensitive to cis/nab pac cytotoxic effects was examined by quantification of burden of copy number alterations and somatic mutations using whole exome sequencing. The post-PIKTOR biopsy tissue from patient 9 was excluded from analysis as it did not pass quality control. Eight of nine patients’ post-PIKTOR biopsies displayed an increase in fraction copy number-altered genome (Fig. 4A and B), and five of nine patients displayed an increase in tumor mutation burden (TMB) (Fig. 4C and D). While most changes to fraction copy-number-altered genome and TMB were within 20% of the pre-PIKTOR levels, a subset of patients displayed a large increase in fraction copy number-altered genome. Of note, responder patients 1 and 6 accounted for 2 of the top 3 largest increases in fraction copy-number altered genome. Two responders, patients 6 and 8, displayed 2 out of the 3 highest tumor mutation burden increases following PIKTOR treatment.

To assess whether major oncogenic signaling pathways were altered following PIKTOR treatment, changes in mutation status of COSMIC cancer gene consensus Tier 1 oncogenes and tumor suppressor genes were assessed (Supplemental Fig. 4). Overall, mutation status of 22 known cancer genes changed between pre- and post-PIKTOR biopsies, occurring in 5 of 10 patients. We did not detect definitive changes that might explain the TMB and copy number alterations observed (results are summarized in Supplemental Information).

### DNA damage repair defects before and after PIKTOR treatment

**Fig. 5:**
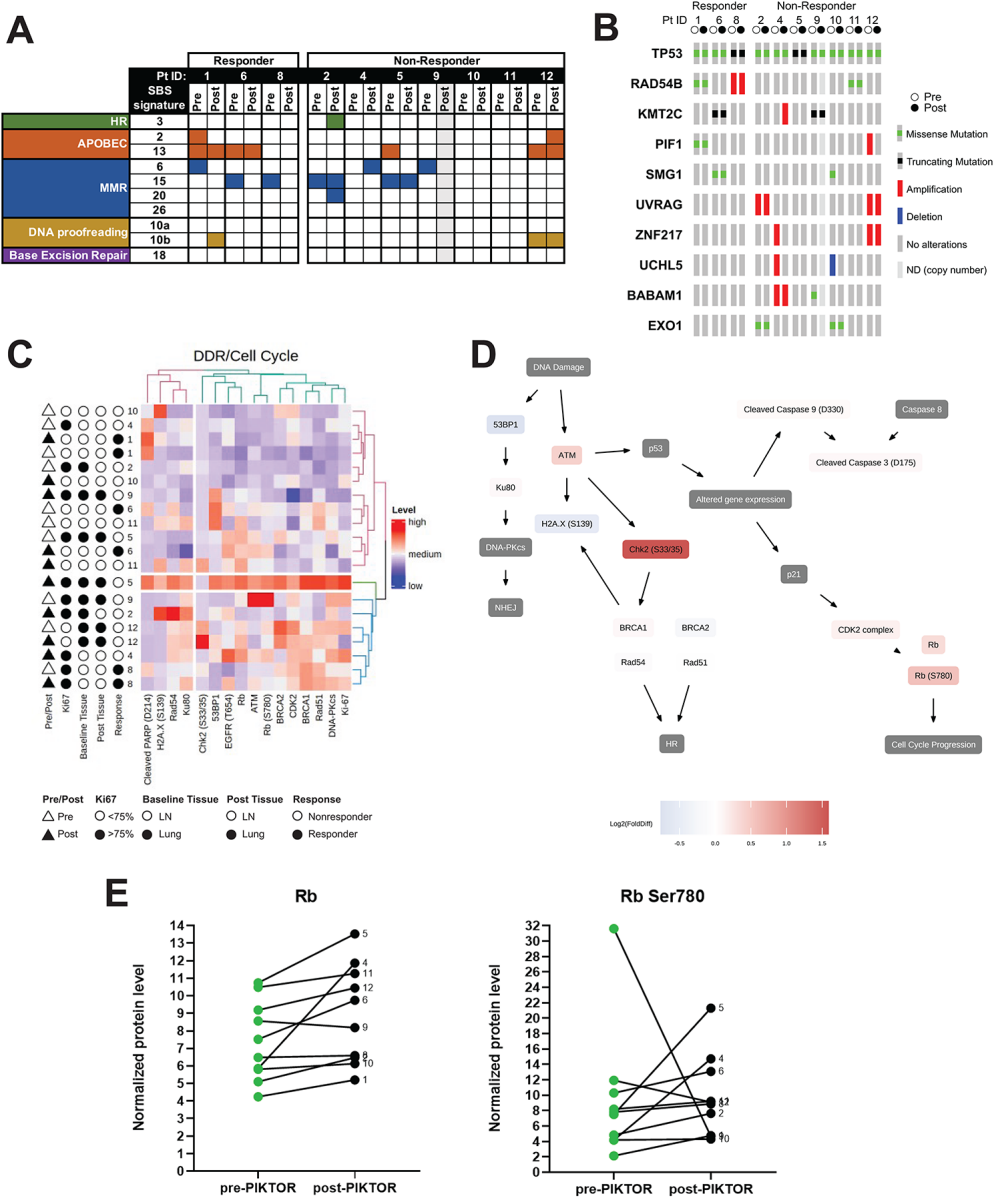
DNA damage repair signatures before and after PIKTOR treatment. **(A)** Mutational signatures of DNA damage repair using COSMIC v3 Mutation Signature. SBS signature ID is highlighted, and presence of DNA damage repair defect is indicated by filled space in table. **(B)** Oncoprint of somatic variants and copy number alterations to genes involved in DNA repair and associated chromatin remodeling. Pre/Post samples are designated by white and black circles, respectively. Variant or copy number alteration is designated as represented in the legend. ND = not determinable for copy number. **(C)** Unsupervised hierarchical clustering levels of DNA damage repair pathway proteins in microdissected lymph node metastasis reveals heterogeneous signaling profiles. Pre and post samples are represented by white and black triangles, respectively. **(D)** Biological signaling pathway superimposed with log_2_ fold changes in average protein levels pre-PIKTOR compared to post-PIKTOR. Patients with lymph node metastasis biopsy specimens both pre and post-PIKTOR were 1, 4, 6, 8,10 and 11. Sample 2 was excluded from analysis because the pre and post biopsy were from different metastatic sites (lung and lymph node). **(E)** Phosphorylation of Rb allows cell cycle progression. Increased levels of phosphorylated Rb protein in responders post PIKTOR treatment may sensitize cells to DNA damaging therapies such as cisplatin. Only patient #6 had a *Rb* gene mutation in the pre-PIKTOR sample

Patients’ tumors may have differential response to PIKTOR treatment based on either baseline or acquired alterations in DDR deficiency. Using mutation signature analysis, genomic signatures of DDR including homologous recombination (HR), APOBEC, mismatch repair (MMR), and DNA proofreading were assessed (Fig. 5A). The most prominent DDR signatures in the overall cohort were MMR (7 of 10 patients) and APOBEC (4 of 10). Interestingly, signatures of defective MMR were lost in all three responders’ lymph node metastases following PIKTOR treatment, suggesting that tumor cells with MMR deficient signature were lost following PIKTOR treatment. Two non-responders’ tumor tissues gained MMR deficiency signatures post-PIKTOR, two others retained it, and non-responder patient 9’s pre-PIKTOR tissue demonstrated the defective MMR signature while the post-PIKTOR tissue could not be assessed. Only one patient’s (non-responder patient 2) tumor gained the HRD signature following PIKTOR treatment, but the pre-and post-PIKTOR biopsies were obtained from lung and lymph node, respectively.

We also examined how DDR genes themselves may have been altered either at baseline or following PIKTOR treatment by examining somatic variants and copy number alterations (Fig. 5B) including key histone modifying and chromatin remodeling genes known to affect DDR [43]. All patients had *TP53* mutations in both pre- and post-PIKTOR biopsies. No known inactivating mutations in DDR genes were observed in patients’ biopsy samples except for patient 1 whose cancer harbored a germline frameshift mutation in *BRCA1* (data not shown) without loss of heterozygosity, suggesting at least partial HR proficiency.

Only a few DDR genes were amongst the differentially expressed genes in our RNA-seq analyses (Supplemental Table 4). The 3 responders had a statistically significant decrease in ZNF365 expression (log_2_FC = -3.00), a homologous recombination repair gene, following PIKTOR treatment, which was not observed in non-responders. Interestingly, global changes in gene expression in DDR pathways were largely absent in our analysis of the pre- and post-PIKTOR tissues (Supplemental Table 4). Given the loss of MMR deficiency signatures in the 3 responders, we looked specifically at changes in gene expression of 22 MMR genes [44], but did not identify changes in expression that would account for the MMR deficiency signature loss, even when nonsignificant trends were considered. This further supports a shift in clonality of following PIKTOR treatment.

### Heterogeneous protein signaling in mTNBC DDR pathway proteins

We assessed the expression of DDR proteins associated with the HR and NHEJ pathways pre- and post-PIKTOR. DDR pathway level mapping directly assesses the HR pathway via BRCA1, BRCA2, Rad51, Rad54 levels, while 53BP1, Ku80, DNA-PKcs, and 53BP1 protein levels reflect activation/inactivation of NHEJ. Unsupervised hierarchical clustering of DDR pathways (Fig. 5C) revealed heterogenous signaling profiles as shown by the lack of clustering by responder/non-responder or pre/post PIKTOR treatment categories. Comparative analysis of the signaling features between responders and non-responders yielded an analogous heterogenous signaling profile. The clustering patterns between patients and high degree of similarity of select DDR proteins such as Ku80 and Rad54 before and after PIKTOR (Supplemental Fig. 5) indicate no obvious global changes in expression of HR and NHEJ proteins post-PIKTOR [45]. We superimposed the protein log_2_-fold change for microdissected tumors metastatic to lymph nodes on a DNA damage response signaling pathway to look for trends in protein nodes post-PIKTOR compared to pre-PIKTOR (Fig. 5D). ATM, Chk2 Ser33/35, and Rb Ser780 were higher in the post-PIKTOR tissues. We noted increased phosphorylated retinoblastoma protein post-PIKTOR in only the metastatic lymph node tissues. Retinoblastoma protein is a tumor suppressor and phosphorylated Rb (Ser780) releases the suppression, promoting cell cycle progression. Increased phosphorylation of Rb protein post- PIKTOR could sensitize cells to DNA damaging therapies such as cisplatin (Fig. 5E). Only patient 6 had a *Rb* gene mutation in the pre-PIKTOR sample. The lack of uniform changes in DNA damage repair protein levels between responder and non-responder groups may be due to different DNA repair mechanisms between patients and/or variation in cellular stress response pathways that exacerbate or hinder DNA repair.

### Immune signature following PIKTOR treatment

**Fig. 6:**
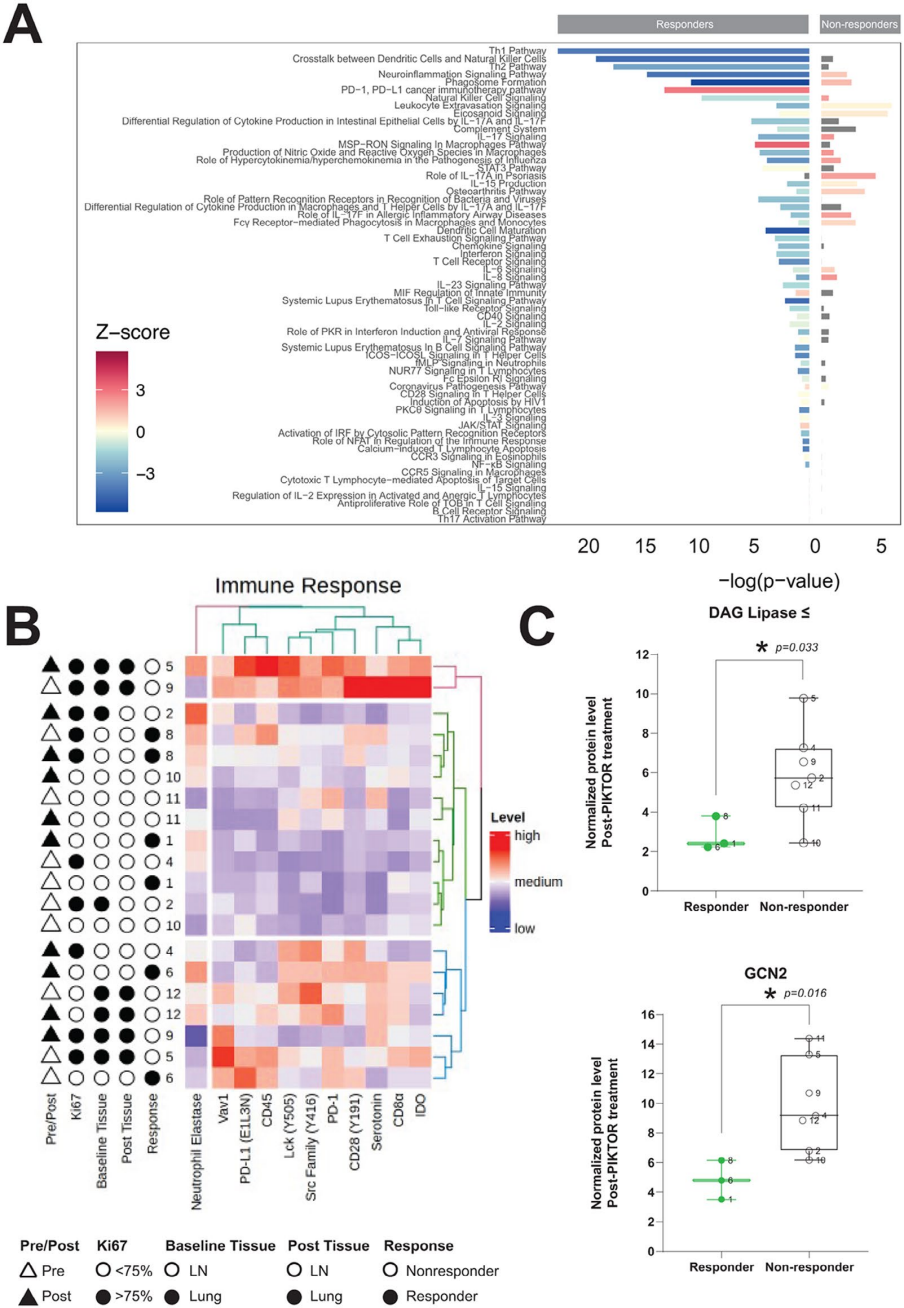
Changes to immune and inflammatory pathways with PIKTOR treatment. **(A)** Canonical pathway enrichment from differential gene expression results between pre- and post-PIKTOR biopsies. DESeq2 results from independent Responder and Non-responder analyses were input into Ingenuity Pathway Analysis, and results from the canonical pathway output is grouped by broader common pathways. Z-score is represented by color, and –log(p-value) is displayed by bar chart height, with values for responders extending to the left of the origin, and values for non-responders to the right. **(B)** Unsupervised hierarchical clustering levels of immune pathway proteins in microdissected lymph node metastasis reveals heterogeneous signaling profiles. Pre and post PIKTOR samples are represented by white and black triangles, respectively. **(C)** Diacylglycerol lipase Beta and General Control Non-derepressible 2 (GCN2) protein levels were significantly decreased in responders compared to non-responders following PIKTOR treatment

To gain insight into why 3 patients had highly durable responses with pembrolizumab following PIKTOR and cis/nab pac, we evaluated protein and gene expression levels of immune signaling pathway components within the TNBCs. Responders displayed substantially decreased immune pathway gene expression post-PIKTOR, including Th1, Th2 dendritic cell/natural killer cell crosstalk, and natural killer cell signaling. Conversely, the PD-1/PD-L1 immune checkpoint pathway was highly increased on gene expression analysis for the non-microdissected post-PIKTOR treatment specimens only in the patients who responded durably to cis/nab pac followed by pembrolizumab (Fig. 6A).

PD-L1 RNA and PD-1 and PD-L1 protein levels were variable before and after PIKTOR (Supplemental Fig. 6). The commonality among the 3 responders is a decrease in CD45 and increase in Lck Y505 protein levels post PIKTOR. CD45 activates lymphocytes by dephosphorylating Lck Y505 [46]. This pattern appears to indicate a lack of activation and downstream signaling in Src family kinases. These data suggest that proteins other than PD-1 and PD-L1 in the microdissected tumor specimens led to a decrease in lymphocyte activation and subsequent downstream proliferation and inflammation signaling (Supplemental Figs. 7 & 8). Unsupervised hierarchical clustering of the entire cohort of microdissected tumor samples for immune and inflammatory proteins showed heterogenous clustering (Fig. 6B).

### Decreased stress response and immune tolerance in responders to pembrolizumab

The DNA damage repair response was heterogeneous among our responder and non-responder groups. We therefore sought to determine if any of the protein biomarkers could potentially be used to identify responders and non-responders. We performed a statistical comparison of all protein levels for all lung and lymph node metastatic specimens (n = 10). Higher expression of DAG Lipaseβ (p < 0.05) and GCN2 (p < 0.05) were found among the non-responder cohort (n = 7) compared to the responder cohort (n = 3) (Fig. 6C). DAG Lipaseβ mediates arachidonic acid metabolic processes in macrophages, modulating the inflammation response [47]. GCN2 regulates protein synthesis and optimal usage of macromolecules by phosphorylating eIF2alpha to downregulate protein synthesis in response to stress [48], an expected consequence of mTOR inhibition. These results suggest that the non-responders’ post-PIKTOR tumor tissues may have initiated the integrated stress response under metabolic stress induced by PIKTOR treatment.

**Fig. 7:**
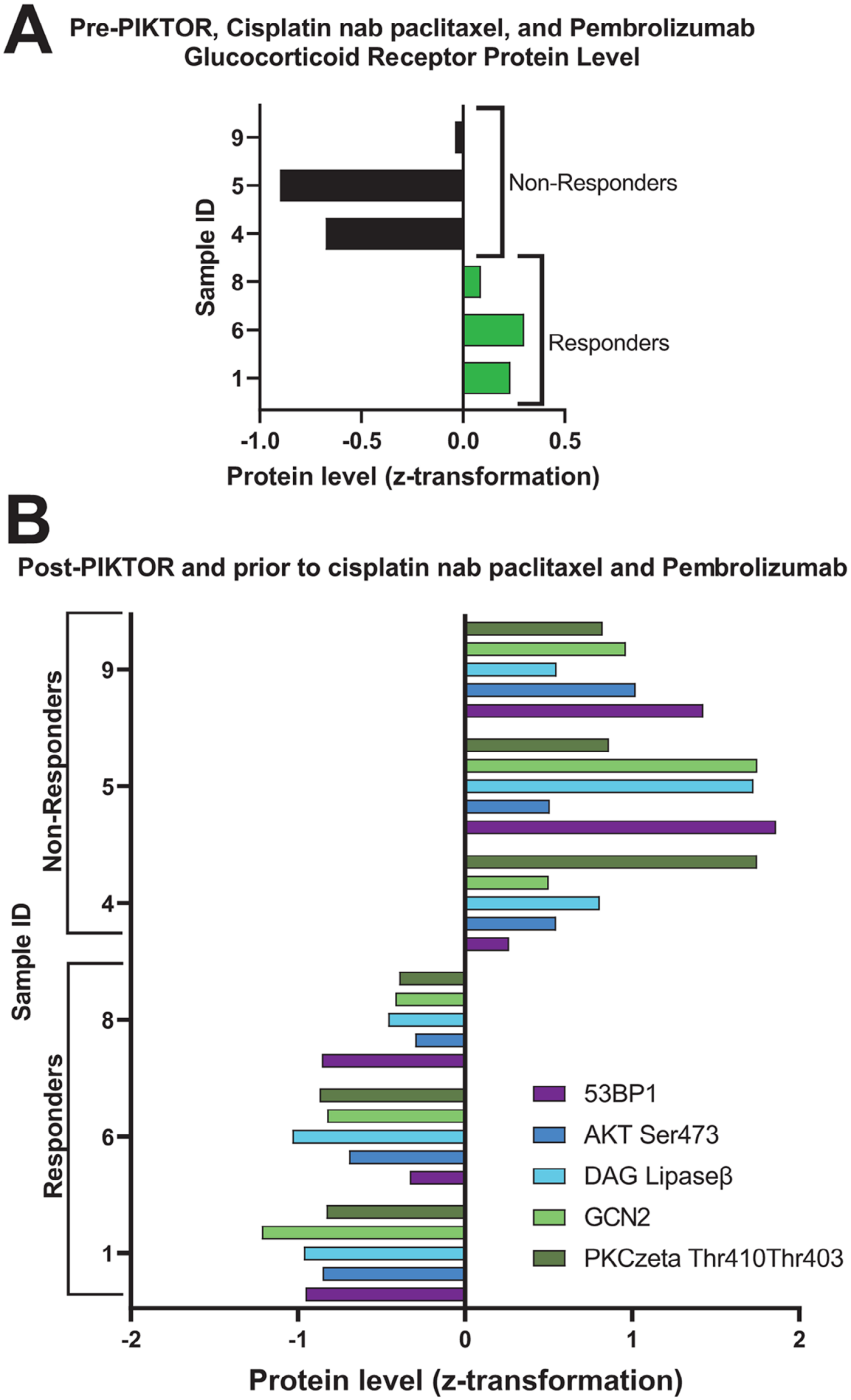
Differential protein levels in metastatic lymph node and lung biopsies for the subset of patients treated with pembrolizumab following PIKTOR and cisplatin nab paclitaxel. Pre-treatment biopsies were obtained prior to the study treatments. Post-PIKTOR biopsies were obtained following PIKTOR treatment, but prior to cisplatin nab paclitaxel and pembrolizumab. **(A)** Glucocorticoid receptor protein levels were higher in biopsies obtained prior to PIKTOR, cisplatin nab paclitaxel, and pembrolizumab for the subset of patients that had a durable response to pembrolizumab. **(B)** Proteins involved in DNA damage repair, proliferation, immune cell activity, and chemotaxis were significantly lower in post-PIKTOR treatment biopsies for patients that responded to pembrolizumab compared to non-responders. Samples 1, 6, 8, and 4 were lymph node metastasis. Sample 5 and 9 were lung metastases

To decipher specific proteins that could potentially be used as biomarkers for likelihood of response to immune checkpoint inhibitors, we compared protein levels only for the patients who received pembrolizumab (n = 3 responders and 3 non-responders (Fig. 7)). Glucocorticoid Receptor protein levels were higher in the lung and lymph node biopsies obtained prior to PIKTOR, cis/nab pac, and pembrolizumab for the subset of patients that had a durable response to pembrolizumab (*p = 0.048*).

We found a set of 5 proteins that were significantly lower following PIKTOR in the patients who had a durable response to pembroluzimab (Fig. 7B). 53BP1, DAG Lipase β, GCN2, AKT Ser473, and PKCzeta Thr410/403 were decreased in the microdissected tumor specimens metastatic to lymph node (patients 1, 6, and 8) post-PIKTOR. 53BP1 and AKT Ser473 help drive DNA repair [49, 50] while GCN2 reduction prevents cells from upregulating metabolic stress responses [51]. DAG Lipase β activity is increased in macrophages and immune cells [52, 53]. PKCzeta Thr410/403 promotes proliferation and invasion [54]. These proteins could potentially serve as biomarkers of response to immunotherapy.

## Discussion

The goals of this pilot trial were to examine the safety and efficacy of combined PIK3CA and TORC1/2 inhibition with PIKTOR, followed by cis/nab pac chemotherapy in patients with metastatic TNBC, and to investigate whether dual inhibition of the PI3K pathway would increase DDR deficiency and tumor immunogenicity. Interestingly, 3 of the 10 patients benefited durably with prolonged response/stable disease with cis/nab pac (patients 1, 6, and 8, with patient 8 having minimal, equivocal nodal progression only on cis/nab pac) followed by single agent pembrolizumab. These 3 patients had platinum-pretreated, nodal-dominant metastatic TNBC (one of the patients also had bone disease and a solitary brain metastasis), two PD-L1 negative and one PD-L1 positive, and would not have been likely to have a durable response to chemotherapy nor to pembrolizumab. A recent trial of the mTOR inhibitor, everolimus, in combination with cisplatin given preoperatively to TNBC patients with residual disease following preoperative anthracycline/taxane showed responses to therapy in patients whose cancers had a *PIK3CA* mutation or a germline PALB2 mutation [55]. None of the 3 responders’ cancers in the PIKTOR/cis/nab pac trial harbored a *PIK3CA* mutation in their pre-PIKTOR biopsies, and patient 1 had a germline *BRCA1* mutation, although this PARP inhibitor-pretreated patient’s pre- and post-PIKTOR biopsies did not demonstrate somatic BRCA1 loss of heterozygosity.

Preclinical studies have demonstrated that PI3K inhibition can enhance antitumor immunity and susceptibility of TNBC to immune checkpoint inhibition [56, 57]. PI3K/AKT/mTOR pathway signaling was not statistically significantly altered in the post-PIKTOR tumor specimens across all patients in our differential gene and protein expression analyses. Pathway analysis using RNA-seq data displayed a trend towards decreased inositol synthesis and metabolism pathways, PI3K signaling, and p70S6K signaling in the 3 responder patients’ post-PIKTOR biopsies (Fig. 3A) but not in the non-responders. PI3K/AKT/mTOR protein pathway signaling in patients with lymph node metastasis (n = 6), did not reveal common, complete pathway up or down modulation for all 6 patients (Fig. 3C and Supplemental Fig. 9). It is possible that the timing of the post-PIKTOR biopsies, at disease progression on PIKTOR, was not an optimal time-point to observe maximal gene or protein inhibition across the entire pathway. Another possibility is compensatory protein pathway signaling in response to PIKTOR treatment may reflect individual patient’s underlying cell signaling pathways in these microdissected tumor samples.

The underlying premise for the trial was that PIKTOR would sensitize TNBC to subsequent DNA damage-inducing therapy by impairing DDR pathways. We calculated tumor mutation burden and fraction of copy number altered genome and observed increases in both measures post-PIKTOR in the responders, consistent with this hypothesis. Mismatch repair deficiency signatures were lost in the post-PIKTOR biopsies from all three responders, compared with none of the non-responders, signifying loss of mismatch repair deficiency. However, we cannot exclude two possibilities: clonal heterogeneity based on sampling variation within the metastatic lymph nodes, and/or that the observed increase in TMB in the responders’ tissues post-PIKTOR may have affected ascertainment of the MMR deficiency signatures, though most other signatures were concordant. We did not observe genomic alterations in DDR genes in responders that could explain the loss of the MMR deficiency signature and increased TMB post-PIKTOR. We may have expected HRD signatures to change more, particularly in the responders, but that was not observed. However, we did see some evidence of change to components of the HR pathway, including strong decreases in ZNF365 expression in responders only after PIKTOR treatment. It is thus possible that signatures of DDR deficiency are complex and masked by the simplification of DDR signatures to common DNA mutation patterns. A further limitation to evaluating these factors is the timing of biopsies, where further pressure from DNA damaging cis/nab pac may have exposed more DNA repair deficiency. However, to avoid complications analyzing potentially necrotic tissue post-cis/nab pac, we opted to do the second biopsy following PIKTOR treatment.

The three patients who responded durably to single agent pembrolizumab after PIKTOR followed by cis/nab pac therapy had high tumor mutational burden in their post-PIKTOR biopsies, a known predictor of benefit from pembrolizumab in metastatic TNBC patients [58, 59]. Pathway analysis of gene expression profiles demonstrated dampening effects of PIKTOR across many immune and inflammatory pathways in the 3 patients who subsequently responded to cis/nab pac followed by pembrolizumab. The observed increase in the canonical PD-1/PD-L1 pathway post-PIKTOR using Ingenuity Pathway Analysis is particularly interesting as this was observed only in the 3 patients who responded durably to post-PIKTOR pembrolizumab. PD-1/PD-L1 pathway activation, but not increased PD-L1 gene expression, was observed in the responders’ cancers post-PIKTOR. PD-1 protein levels were not substantially changed in the responders’ cancers post-PIKTOR, but did increase in several of the non-responders’ cancers post-PIKTOR (Supplemental Fig. 6). The dichotomy in gene expression and protein results could be due to the use of heterogeneous tissue (not microdissected) for gene expression analysis versus microdissected tumor tissue for proteomic analysis.

Functional proteomic analysis of immune and inflammation pathways showed changes in regulators of immune cell activation. CD45 is a positive regulator of T cell antigen receptor (TCR)- and B cell antigen receptor (BCR)-mediated signaling activation and lymphocytic development [60]. Elevated levels of phospho-Lck (Y505) were observed in 8 of the 10 patients’ cancers post-PIKTOR, including in the 3 responders (Fig. 3C). Lck activates T cell receptor signaling [61] while phosphorylation of Lck at Tyrosine 505 decreases catalytic activity. Increased levels of Lck (Y505) post-PIKTOR suggest decreased T cell receptor signaling in the microdissected cancers that had metastasized to lymph nodes. Nonetheless, the 3 durable responses suggest that T cell signaling may have been subsequently activated by cis/nab pac/pembrolizumab in these patients.

We found patients who had a durable response to pembrolizumab following PIKTOR and cis/nab pac [1, 6, 8] had higher levels of Glucocorticoid Receptor in the pre-PIKTOR biopsies. Glucocorticoid Receptor activation has been shown to induce apoptosis in lymphocytes, whereas it promotes proliferation in breast epithelium [62]. Elevated Glucocorticoid Receptor has also been shown in TNBC to correlate with chemotherapy resistance [62]. Our patient cohort was heavily pre-treated prior to initiating PIKTOR therapy. The elevated Glucocorticoid Receptor level in pre-PIKTOR specimens may reflect chemotherapy resistance, or proteomic signaling changes inherent in lymph node metastasis due to microenvironment signaling [63, 64].

DNA damage repair protein signaling involves intersecting and discreet protein pathways. We noted variation of DNA pathway repair protein levels between patients that could be due to the underlying genomic alterations as well as differences in protein signaling due to the patient’s prior therapies (Table 1). Proteins involved in DNA damage repair (53BP1), proliferation and chemotaxis (AKT Ser473, PKCzeta Thr410/403), immune cell activity (GCN2, DAG Lipase β) were lower in responders compared to non-responders in the post-PIKTOR specimens. These proteins represent various cell signaling networks that collectively could contribute to the observed durable immunotherapy responses. 53BP1 promotes NHEJ and acts as a scaffold for recruiting DNA damage response proteins. In cell models of ionizing radiation damage, 53BP1 was shown to bind p53 to facilitate G_2_-M check point arrest and phosphorylate Chk2 and BRCA1, activating DNA damage repair [49]. Bouwman et al. [65] showed that a subset of TNBC patients with 53BP1 positive cells had worse overall survival.

Lower protein levels of GCN2 following PIKTOR treatment in the responders were likely a by-product of PI3K and mTOR pathway inhibition, thus disrupting energy acquisition and metabolism regulation (Figs. 6C and 7). GCN2 initiates the Integrated Stress Response in reaction to depleted amino acid levels. An increase in GCN2 stalls protein translation by inhibiting eIF2alpha [66–68]. This inhibition of protein production allows the cell to upregulate autophagy or modulate other feedback loops to maintain homeostasis. Increased GCN2 levels have been shown to promote immunologic tolerance, thus making immunotherapy ineffective or less effective [51, 69, 70].

Metabolic cell stress could lead to a nucleotide pool imbalance, thus affecting DNA repair efficiency and genomic stability [71, 72]. Diehl et al. recently reported that an imbalanced nucleotide pool inhibits cell proliferation and cells use replicative stress signaling via ATR, rather than autophagy, to maintain cell growth [73]. We did not find upregulation of autophagy proteins (LC3B, mTOR, and AMPK) post-PIKTOR and Cleaved PARP1 and H2AX Ser139 levels, markers of apoptosis and base excision repair respectively, were not significantly different between responder and non-responder groups (Fig. 5C-D). However, we did note a dual decrease in protein levels of 53BP1 and H2AX Ser139 post-treatment, which are independent of ATM mediated DNA repair that could affect the efficiency of single strand break or base excisional repair [71, 72].

Nicotinamide adenine dinucleotide (NAD^+^), and its reduced form NADH, are crucial cofactors for oxidation/reduction enzymes that regulate cell metabolism. NAD is synthesized in the kynurenine pathway which uses L-tryptophan as a precursor for synthesis of NAD [74]. Sirtuin family deacetylases (SIRTs), poly(ADPribose) polymerases (PARPs) consume NAD^+^ [75, 76]. Cellular stress resulting in increased tryptophan degradation via indoleamine 2,3-dioxygenase (IDO) could ultimately alter the NAD pool and the activity of sirtuins and PARP, ultimately changing the efficiency of DNA repair. Further analysis of tryptophan metabolism, ATR, sirtuins, and PARP pathway signaling could provide a better understanding of DNA repair kinetics in PIKTOR treated breast tumors.

Our results suggest that the responders’ cancers were not able or as efficient at upregulating stress responses and thus the tumor cells were more susceptible to cell death under metabolic stress. Immunotherapy in the metastatic LN niche may be effective due to a high proportion of T cells in the LN. Increased tumor proliferation depletes the local nutrient pool. GCN2 can be activated by amino acid deprivation or reduced tryptophan pool (through the serotonin, kyneurine, tryptophan-IDO pathway) [77, 78]. Disrupted energy metabolism followed by cis/nab-pac chemotherapy would further enhance cellular stress. The compounded effects of PIKTOR and chemotherapy on altered cellular metabolism could potentially lead to successful immune checkpoint inhibitor blockade if the tumor is unable to upregulate stress and DNA damage repair proteins [79, 80].

Limitations of this pilot study include small sample size, analysis of microdissected tissue for proteomic studies versus heterogeneous whole tissue samples for genomic studies, and variability in tumor biopsy sites pre- and post-PIKTOR, i.e., lymph node and lung in one patient. However, this study does show the ability to procure serial research biopsies and the prognostic and theranostic utility of multi-omic analysis of TNBC patients with advanced metastatic disease. Additional interpretations on DDR signatures, immune response, stress pathways and others would have been aided by an extended set of serial research biopsies following cis/nab-pac and immunotherapies. However, these research biopsies were not originally in the trial design and are unavailable for analysis. Although only a subset of patients demonstrated clinical benefit from the sequential administration schema, it is likely that combined inhibition of PI3K and TORC1/2 with PIKTOR would be too toxic to combine with therapeutic doses of cytotoxic agents.

In conclusion, inhibition of PIK3CA and TORC1/2 with oral PIKTOR prior to treatment with cis/nab pac in metastatic TNBC patients is safe, and a subset of patients with platinum-pretreated metastases had multi-year disease control with sequential treatment with PIKTOR, cis/nab pac, then pembrolizumab. In the patients with durable responses, metastatic nodal disease assessed following disease progression on PIKTOR showed increased TMB, loss of MMR deficiency signature, decreased immune/inflammation pathway protein levels for DNA damage, cellular stress, and proliferation compared to their pre-PIKTOR nodal metastatic disease. At this time, PIKTOR is no longer being evaluated in mTNBC patients, however, trials including other PI3K/AKT pathway inhibitors, alpelisib and capivasertib, in combination with cytotoxic therapy in mTNBC patients are ongoing. Our findings may help inform analyses in these trials seeking biomarkers of therapeutic benefit.

## Electronic supplementary material

Below is the link to the electronic supplementary material.

Supplementary Material 1

Supplementary Material 2

Supplementary Material 3

Supplementary Material 4

Supplementary Material 5

Supplementary Material 6

Supplementary Material 7

Supplementary Material 8

Supplementary Material 9

Supplementary Material 10

Supplementary Material 11

Supplementary Material 12

Supplementary Material 13

Supplementary Material 14

Supplementary Material 15

Supplementary Material 16

Supplementary Material 17

## Data Availability

The genomic data generated in this study are not publicly available due to patients not being consented for genomic data sharing but are available upon reasonable request from the corresponding author. Somatic variants called in whole exome sequencing analysis are summarized in Supplemental Table 3. Differential expression analyses are summarized in Supplemental Table 4. RPPA data are available in Supplemental Table 5.

## List of abbreviations

AR: Androgen receptor
ATP: Adenosine 5’ triphosphate
BCR: B cell antigen receptor
BSA: Bovine serum albumin
Cis/nab pac: Combined cisplatin and nab paclitaxel
cis: Cisplatin
CNV: Copy number variant
COSMIC: Catalog of Somatic Mutations in Cancer
DDR: DNA damage repair
DNA-PKcs: DNA-dependent protein kinase catalytic subunit
ERα: Estrogen Receptor alpha
FA: *Fanconi Anemia*
GCN2: General control non-depressible 2
H&E: Hematoxylin-eosin
HRD: Homologous recombination deficiency
IPA: Ingenuity Pathway Analysis
LCM: Laser capture microdissection
LN: Lymph node
MMR: Mismatch repair
mTNBC: Metastatic triple negative breast cancer
mTOR: Mechanistic target of rapamycin
nab pac: Nab paclitaxel
NHEJ: Non-homologous end joining
OCT: Optimal cutting temperature
PAM: PI3K/AKT/mTOR
PD: Progressive disease; disease progression
PI3K: Phosphoinositide 3-kinase
PI3Kα: Phosphoinositide 3-kinase alpha isoform
PIKTOR: Combined TAK-288 and TAK-117
PR: Partial response
Rb: Retinoblastoma
RPPA: Reverse phase protein array
SD: Stable disease
SNV: Somatic variant
SV: Structural variant
TAK-117: PI3Kα inhibitor
TAK-288: TORC1/2 inhibitor
TCEP: Tris(2-carboxyethyl)phosphine
TCR: T cell antigen receptor
TGEN: Translational Genomics Research Institute
TMB: Tumor mutation burden
TNBC: Triple negative breast cancer
T-PER™: Tissue Protein Extraction Reagent
