# Supplementary material for: Pilot clinical trial and phenotypic analysis in chemotherapy-pretreated, metastatic triple-negative breast cancer patients treated with oral TAK-228 and TAK-117 (PIKTOR) to increase DNA damage repair deficiency followed by cisplatin and nab paclitaxel
Source: Biomark Res. 2023 Jul 25;11:73. doi: 10.1186/s40364-023-00511-7 (PMC10369813; doi:10.1186/s40364-023-00511-7)
Supplement: Supplementary file 4 — Supplementary Material 4 [file 40364_2023_511_MOESM4_ESM.docx]

**Supplementary Information**

**Title:** Pilot clinical trial and phenotypic analysis in chemotherapy-pretreated, metastatic triple-negative breast cancer patients treated with oral TAK-228 and TAK-117 (PIKTOR) to increase DNA damage repair deficiency followed by cisplatin and nab paclitaxel

**Authors:** Jessica D. Lang, Tuong Vi V. Nguyen, Maren K. Levin, Page E. Blas, Heather L. Williams, Esther San Roman Rodriguez, Natalia Briones, Claudius Mueller, William Selleck, Sarah Moore, Victoria L. Zismann, William P.D. Hendricks, Virginia Espina, and Joyce O’Shaughnessy

# Supplemental Figure Legends

**Supplemental Figure 1. Canonical pathway enrichment from differential gene expression results between pre- and post-PIKTOR biopsies.** DESeq2 results from independent Responder and Non-responder analyses were input into Ingenuity Pathway Analysis, and results from the canonical pathway output is grouped by broader common pathways. Z-score is represented by color, and –log(p-value) is displayed by bar chart height, with values for responders extending to the left of the origin, and values for non-responders to the right.

**Supplemental Figure 2. Proteins statistically different pre and post PIKTOR treatment for all samples (n=10).** Triple negative breast tumor cells were metastatic to lymph node or lung.

**Supplemental Figure 3. Proteins statistically different pre and post PIKTOR treatment for mTNBC tumors metastatic to lymph node and lung.** Lymph node metastasis samples were 1, 6, 8, 4, 10, and 11. Lung metastasis samples were 5, 9, and 12. Sample 2 was excluded because the pre-PIKTOR (lung) and post-PIKTOR (lymph node) biopsy tissue were different.

**Supplemental Figure 4. Oncoprint results of changed variants in known oncogenes and tumor suppressor genes between pre- and post-PIKTOR biopsies.** The COSMIC Tier 1 cancer gene consensus was used to designate genes as oncogenes (red gene name), tumor suppressor genes (blue gene name), or both (gradient red/blue gene name). Variants changed between pre- and post-PIKTOR biopsies were categorized based on gain, loss, or mixed gain & loss in patients. Pre/Post samples are designated by white and black circles, respectively. Variant or copy number alteration is designated as represented in the legend. ND = not determinable for copy number.

**Supplemental Figure 5. DNA Damage Response and DNA damage repair pathway proteins. Percent difference in protein levels pre and post PIKTOR. Percent difference=(post-pre)/pre*100.**

**Supplemental Figure 6. Immune checkpoint signatures pre and post PIKTOR treatment.**

(Top panel) Three specimens (1, 10, 11) did not show a correlation between RNA and protein levels for PD-L1 following PIKTOR treatment. This lack of concordance could be due to differences in biopsy tumor content, heterogeneous tissue (RNA) versus microdissected tumor (protein), or post-translational modifications of the protein. (Bottom panel) PD-1 and PD-L1 protein levels were not significantly different among the responder and non-responder groups following PIKTOR treatment. PD-L1 increased post-PIKTOR in patient 1, and decreased in patients 6 and 8. (* indicates patients that received pembrolizumab post PIKTOR treatment).

**Supplemental Figure 7. Immune pathway proteins. CD45 protein levels decreased post-PIKTOR in the 3 mTNBC patients with durable response following treatment PIKTOR, cisplatin, nab paclitaxel, and pembrolizumab. Percent difference in protein levels pre and post PIKTOR. Percent difference=(post-pre)/pre*100.**

**Supplemental Figure 8. Tryptophan metabolism pathway and integrated stress response proteins. Percent difference in protein levels pre and post PIKTOR. Percent difference=(post-pre)/pre*100.**

Supplemental Figure 9. PI3Kinase, AKT, mTOR pathway proteins. Percent difference in protein levels pre and post PIKTOR. Percent difference=(post-pre)/pre*100.
