# Supplementary material for: Pilot clinical trial and phenotypic analysis in chemotherapy-pretreated, metastatic triple-negative breast cancer patients treated with oral TAK-228 and TAK-117 (PIKTOR) to increase DNA damage repair deficiency followed by cisplatin and nab paclitaxel
Source: Biomark Res. 2023 Jul 25;11:73. doi: 10.1186/s40364-023-00511-7 (PMC10369813; doi:10.1186/s40364-023-00511-7)
Supplement: Supplementary file 15 — Supplementary Material 15 [file 40364_2023_511_MOESM15_ESM.docx]

**Supplementary Information**

**Title:** Pilot clinical trial and phenotypic analysis in chemotherapy-pretreated, metastatic triple-negative breast cancer patients treated with oral TAK-228 and TAK-117 (PIKTOR) to increase DNA damage repair deficiency followed by cisplatin and nab paclitaxel

**Authors:** Jessica D. Lang, Tuong Vi V. Nguyen, Maren K. Levin, Page E. Blas, Heather L. Williams, Esther San Roman Rodriguez, Natalia Briones, Claudius Mueller, William Selleck, Sarah Moore, Victoria L. Zismann, William P.D. Hendricks, Virginia Espina, and Joyce O’Shaughnessy

# List of Supplemental Tables

**Supplemental Table 1.** DNA Damage Repair (DDR) genes

**Supplemental Table 2.** RPPA antibody list, protein name, and gene name.

**Supplemental Table 3.** Variants by patient

**Supplemental Table 4.** DESeq results

**Supplemental Table 5.** Reverse Phase Protein Array (RPPA) normalized and z-transformation protein levels.

**Supplemental Table 6.** Adverse events for PIKTOR therapy.
